# Spirituality in the Context of Well-being. Evaluation of the Psychometric Properties and Added Value of the Spiritual Attitude and Involvement List Short Form (SAIL-SF)

**DOI:** 10.1007/s10902-023-00640-8

**Published:** 2023-03-05

**Authors:** E. T. Bohlmeijer, L. Frielingsdorf, J. T. Kraiss, E. de Jager-Meezenbroek, A. Visser, P. M. ten Klooster

**Affiliations:** 1 Department Technology of Human and Institutional Behavior, University of Twente, Drienerlolaan 5, 7522 NB Enschede, The Netherlands; 2 ACT Therapie Utrecht, Utrecht, The Netherlands; 3 Faculty of Theology and Religious Studies, University of Groningen, Groningen, The Netherlands

**Keywords:** Spirituality, Well-being, Ability to adapt, Questionnaire, Short-form

## Abstract

There is growing evidence that spiritual well-being is positively associated with adaptive coping and health. The Spiritual Attitude and Involvement List (SAIL) was developed to measure a sense of connectedness to oneself, the environment and the transcendent as a universal experience. The aim of the current study was to develop a short form of the SAIL (SAIL-SF). A factor analytic approach was adopted to select the items for the SAIL-SF based on earlier studies among nurses (n = 458) and cancer patients (n = 445). The dimensionality, factor-loadings, internal consistency, construct validity and incremental validity of the final SAIL-SF were then evaluated in a new sample of adults (n = 225) participating in a trial assessing a positive psychology intervention. The first study yielded seven items, each representing one of the dimensions of the original SAIL: meaningfulness, trust, acceptance, caring for others, connectedness with nature, transcendent experiences, and spiritual activities. The seven items represented a single meaningful factor in both samples and the factor loadings of the items were adequately high. In the second study, a good fit across the various model indices was found and all items had adequately high factor loadings in a strict unidimensional confirmatory factor model and demonstrated good internal consistency. The SAIL-SF explained 7% of variance in ability to adapt above and beyond emotional, psychological, and social well-being. The current study shows that the SAIL-SF has good psychometric properties, and that spiritual well-being has a unique contribution to the ability to adapt in comparison with other types of well-being.

In the past decades there has been a growing interest in mental well-being. Two traditions of well-being are often distinguished: hedonic well-being and eudaimonic well-being (Ryan & Deci, 2001; Waterman, 1993). Hedonic well-being can be equated with positive emotional functioning (Diener et al., 1999; Keyes, 2002). It is linked to being able to ‘take pleasure from our senses’, such as enjoying food, music, the smell of flowers and sex. The concept of eudaimonia dates back to Aristotle, who believed that the essential element of a good life was the realization of one’s own potential, as opposed to subjective happiness (Waterman, 1993). Ryff (1989, 1991) has been influential in operationalizing the psychological dimension of eudaimonic well-being. She found six elements of positive functioning that make up psychological well-being: self-acceptance, purpose in life, autonomy, positive relationships with others, environmental mastery and personal growth. Each of these elements is important for the realization of one’s potential and living a fulfilled life. In addition to individual functioning as operationalized by Ryff, it has been argued that optimal social functioning is also an essential aspect of eudaimonic well-being (Keyes, 1998). Social functioning involves social engagement and societal functioning. Optimal well-being or flourishing comprises both hedonic well-being, i.e. the presence of emotional well-being as an indicator of feeling well, and eudaimonic well-being with psychological and social well-being as indicators of living well and meaningfully (Keyes, 2002).

However, it has been argued that spirituality is an additional essential component of eudaimonic well-being (Moberg, 1971; van Dierendonck, 2012). Numerous definitions of spirituality have been developed (de Brito Sena, 2021). For example, Koenig et al. (2001) defined spirituality as 'the personal quest to understand end-of-life issues, their meaning, and relationships with the sacred or transcendental that may or may lead to the development of religious practices or religious community'. In comparison, Puchalski et al., (2009, p. 867) defined spirituality more broadly 'as the aspect of humanity that refers to the way in which individuals seek and express meaning and purpose and the way they experience their connectedness to the moment, to self, to others, to nature, and to the significant or sacred'. Despite the variety in definitions of spirituality, most deal with the ultimate goal in life, the experience of a transcendent dimension that gives meaning to existence and the capacity to experience the sacred (Giacalone & Jurkiewicz, 2003). Spirituality is often associated with living by one's inner truth to produce positive attitudes and relationships in one's life (Hawley, 1993). Fisher (2011) states that spirituality alters peoples’ perceptions concerning quality of life and meaning in the personal, communal, environmental, and transcendental domains. As such, spirituality can be considered an inner resource that directly contributes to a sense of purpose in life, resilience, adaptive coping, and well-being (van Dierendonck, 2012). There is a growing body of studies finding positive associations between spirituality on the one hand and well-being, resilience and adaptive coping on the other (e.g., Bai & Lazenby, 2015; Baldacchino & Draper, 2001; Schwalm et al., 2022; Stewart & Yuen, 2011; Visser et al., 2010). Where psychological well-being can be seen as the extent to which one is realizing one's potential and social well-being as optimal societal functioning, spiritual well-being can be considered the extent to which one is living in direct connection with the self as consciousness, with nature and others and with God or transcendent dimension of life (de Jager Meezenbroek et al., 2012b; Moberg, 1971; van Dierendonck, 2012). Spirituality strengthens the purpose in life and meaning dimensions of the good life, from a transpersonal perspective.

Various instruments measuring spirituality and spiritual well-being have been developed, such as the Spiritual Wellbeing Scale of the Functional Assessment of Chronic Illness Therapy (FACIT-Sp-12) (Brady et al., 1999), the Mental, Physical, and Spiritual Wellbeing Scale (SWB) (Vella-Brodrick et al., 1995), the Spirituality Assessment Scale (SAS) (Howden, 1992) and the Spiritual Transcendence Scale (STS) (Piedmont, 1999). For example, the FACIT-Sp, one of the most used spiritual well-being scales, was developed to measure spiritual well-being in people with chronic illnesses. Based on earlier work by Mickley et al. (1992) and Larson et al (1998), three components of spiritual well-being are measured: a personal search for meaning and purpose in life, connection with a transcendent dimension of existence, and the experiences and feelings associated with that search and that connection (Peterman et al., 2002). More recently, de Jager Meezenbroek and colleagues (2012a) developed a questionnaire aiming at the conceptualization of a broad perspective on spiritual attitudes, in such a way that also non-religious individuals with occasional spiritual experiences are sufficiently represented. This perspective is relevant because in the past decades the conceptualization of spirituality has shifted in a majority of western populations. Whereas the traditional view of spirituality involved the recognition of a spiritual realm or higher reality outside of the individual, the notion of spirituality has moved from the position of being externally grounded and objective to internally created and subjective (Hill & Pargament, 2003). De Jager Meezenbroek and colleagues (2012a, p. 142) therefore defined spirituality as “[…] one’s striving for and experience of connectedness with the essence of life, which encompasses three main dimensions: connectedness with oneself, connectedness with others and nature, and connectedness with the transcendent”. Religion may share these properties but refers to a rather institutionalized, traditional exhibition of spirituality (Powell, Shababi, & Thoresen, 2003). Consequently, spirituality refers to broader and more subjective perceptions as compared to religiosity (Fisher, 2011; Lucchetti et al., 2015).

The Spiritual Attitude and Involvement List (SAIL) measures spirituality as a seven-dimensional construct, including *meaningfulness, trust, acceptance, caring for others, connectedness with nature, transcendent experiences* and *spiritual activities* (de Jager Meezenbroek et al., 2012a). A psychometric investigation of the 26-item scale demonstrated sufficient fit of the correlated seven-factor measurement model of the SAIL across a variety of samples of students, healthy populations and cancer patients. The subscales were moderately to strongly correlated with corrected SAIL total scores. Moreover, convergent and discriminant validity were supported, and good internal consistency and adequate test–retest reliability of the different subscales were observed across the different samples. A study by Visser et al. (2017) also revealed a good fit for the measurement model of the SAIL and good internal consistency of the subscales in cancer patients. More recently, a Polish version of the SAIL showed satisfactory psychometric properties and construct validity in a sample of nurses. However, in this study exploratory factor analysis resulted in a six-dimensional model in which the *meaningfulness* and *caring for others* dimensions were merged (Deluga et al., 2020).

Despite its good measurement properties, the length of the SAIL may limit its use in both research and practice, especially if it needs to be used in combination with other instruments or administered repeatedly. Long questionnaires can be perceived as burdensome by participants, resulting in a variety of response effects such as a tendency to select rather identical response options with increasing item numbers (Herzog et al., 1981) and a decrease in motivation (Bradburn et al., 2004). The development of a short form of the SAIL that retains its broad content validity is likely to facilitate its use in both clinical and population studies. This is highly relevant as it has been noted that spiritual well-being is still somewhat understudied in clinical and population studies in comparison to emotional and psychological well-being (van Dierendonck, 2012).

The development of a short form of the SAIL (SAIL-SF) will also facilitate the direct assessment of the added value of spirituality in comparison to other dimensions of well-being. One often used instrument for well-being is the Mental Health Continuum–Short Form (MHC-SF) that was first introduced by Keyes (2005). This instrument assesses theoretically derived aspects of emotional, psychological and social well-being, with one item for each aspect of the three dimensions. Research has consistently found support for the three-factor structure, with excellent reliability and construct validity of the MHC-SF in various healthy and clinical samples across the world, such as the United States (Robitscheck & Keyes, 2009), the Netherlands (de Vos et al., 2019; Franken et al., 2018; Lamers et al., 2011), South Africa (Keyes et al., 2008), and Iran (Joshanloo et al., 2013). There is substantial evidence that both emotional and psychological well-being contribute to resilience and adaptation (Diener et al., 1999; Fredrickson, 1998, 2001; Huppert, 2009; Ryff, 2014). There is also growing evidence for a positive association between spiritual well-being and resilience (e.g., Coppola et al., 2021; Schwalm et al., 2022; Stewart & Yuen, 2011). However, a direct comparison between the various dimensions of well-being has not been conducted. By regressing a generic measure of adaptation on both the three dimension of the MHC-SF and the SAIL-SF, the incremental validity of the SAIL-SF can be assessed. If indeed spiritual well-being is a vital dimension of well-being, it can be expected that it explains unique variance in the ability to adapt over emotional, psychological, and social well-being.

The aim of the present study was first to systematically develop a reliable short form of the Spiritual Attitude and Involvement List (SAIL-SF) that retains all seven aspects of spiritual well-being based on earlier studies with the SAIL in various populations. Secondly, the factor structure, the reliability and the construct and incremental validity of the SAIL-SF were assessed in a new sample of people participating in a trial assessing the effects of a positive psychology app.

## Method

The first goal of the present study was to develop a reliable short form of the SAIL that preserved its content validity by, similar to the development of the MHC-SF, including one item from each of the seven original aspects (subscales) of spiritual well-being as observed and defined by de Jager Meezenbroek et al. (2012a). For this, secondary data analyses were performed on data from nurses (Van Leeuwen et al., 2015) and cancer patients (Visser et al., 2017) that previously completed the original 26-item SAIL to consider both individuals from a clinical population as well as a non-clinical population. The resulting 7-item SAIL-SF was subsequently administered in a general population sample of people participating in a trial assessing the effects of a positive psychology app.

### Participants

#### Sample 1

The sample from the study by Van Leeuwen and Schep-Akkerman (2015) consisted of 458 healthcare workers (84.5% female) employed in three different nursery settings who completed the SAIL (hospital care: *n* = 197, mental health care: *n* = 152, and home care: *n* = 87). The sampling strategy specifically aimed at qualified nurses who had completed their job training and were employed for the previous 5 years at least. The vast majority of participants were female (84.5%) and more than half (51.2%) of them were younger than 41 years of age, with an additional 43.2% being aged between 41 and 60. Most participants considered themselves Christian (51.6%), whereas around thirty percent stated they were atheistic (12.2%), agnostic (2.0%) or not religious (15.3%).

#### Sample 2

The second sample from the study by Visser et al. (2017) included 445 adult patients with various forms of cancer recruited from four hospitals and two radiotherapy institutions in the Netherlands. Similar to the nurses' sample, the majority of participants were female (72.8%). Participants’ ages ranged between 24 and 84 years with a median age of 59 years. Almost half of the participating patients (49.7%) considered themselves as being spiritual, while 52.5% reported being religiously minded*.*

#### Sample 3

The validation sample consisted of people from the general population who perceived stress or reduced well-being and who participated in the Training in Positivity (TiP) study, a randomized controlled trial assessing the effects of a positive psychology app. The trial was registered on ClinicalTrials.gov (number: NCT05292560). At baseline, participants completed a battery of online questionnaires measuring amongst others well-being, anxiety, depression, positive reinterpretation and growth coping, and perceived ability to adapt. The 7-item SAIL-SF was also administered at baseline. In total, 225 people (85.8% female), mostly living in the Netherlands (85.8%) or Belgium (9.3%) and with a mean ± SD age of 43.0 ± 14.6 years, completed the measurements. The majority of participants had finished higher vocational education or university (72.4%) and were in paid employment (56.4%) or self-employment (12.9%).

### Measurements

Next to demographical questions, the following questionnaires were used to validate the psychometric properties of the SAIL-SF in sample 3. All questionnaires were administered in Dutch.

#### Spiritual Well-Being

The original 26-item Spiritual Attitude and Involvement List (SAIL; de Jager Meezenbroek et al., 2012a) measures spiritual well-being on seven subscales: (1) meaningfulness, (2) trust, (3) acceptance, (4) caring for others, (5) connectedness with nature, (6) transcendent experiences, and (7) spiritual activities. On a 6-point Likert scale ranging from 1 (*not at all*) to 6 (*to a very high degree*), respondents rate in how far they think specific statements about spiritual well-being apply to themselves (e.g. “*I know what my position is in life*”). Higher scores indicate higher scores of spiritual well-being. The original Dutch SAIL has shown good psychometric properties in non-clinical and clinical groups (de Jager Meezenbroek et al., 2012a, 2012b; Visser et al., 2017). The 7-item SAIL-SF developed based on the results from samples 1 and 2 was administered in the current sample.

#### Mental Well-Being

The 14-item Mental Health Continuum-Short Form (MHC-SF; Keyes et al., 2008; Lamers et al., 2011) was used to assess mental well-being on three dimensions: emotional (3 items), social (5 items), and psychological well-being (6 items). Participants rated the frequency of feelings in the past month on a 6-point Likert scale, ranging from 0 (*never*) to 5 (*every day*). Higher mean scores are indicative of more mental well-being. The Dutch MHC-SF has shown to be reliable in the general population (Lamers et al., 2011) and in clinical populations (De Vos et al., 2019; Franken et al., 2018). Internal consistency in the current study was excellent for the total scale (ω = 0.92) and acceptable to good for the subscales (ω = 0.76–0.86).

#### Coping and Ability to Adjust

Positive reinterpretation as coping style was measured with the 2-item subscale positive reinterpretation and growth of the Brief-COPE (Carver, 1997). Participants were asked how often they engage in specific coping styles, with the items being answered on a 4-point Likert scale ranging from 1 (*I haven’t been doing this at all*) to 4 (*I’ve been doing this a lot*). Higher total scores indicate more frequent use of the coping style positive reinterpretation. Internal consistency in the current study was good (ω = 0.87).

Ability to adjust was measured with the Generic Sense of Ability to Adapt Scale (GSAAS; Franken et al, 2022). The GSAAS contains ten statements referring to the degree to which an individual is able to adapt to stressful life situations (e.g. “*I can deal well with setbacks*”). The items are rated on a 5-point Likert scale ranging from 1 (*not at all*) to 5 (*totally*). Total higher scores indicate more ability to adapt. The Dutch GSAAS has shown good psychometric properties (Franken et al., 2022). Internal consistency in the current study was excellent (ω = 0.92).

#### Psychopathological Symptoms

Depression was assessed using the 9-item Patient Health Questionnaire (PHQ-9; Kroenke et al., 2001). The PHQ-9 assesses the presence of symptoms of depression in the past 2 weeks. Items are scored on a 4-point Likert scale ranging from 0 (*not at all*) to 3 (*almost every day*). Higher total scores are indicative of more severe symptoms. The Dutch version of the PHQ-9 has been validated before in patients with coronary heart disease (van der Zwaan et al., 2016). Internal consistency in the current study was acceptable (ω = 0.77).

Symptoms of anxiety were assessed with the General Anxiety Disorder Questionnaire (GAD-7; Donker et al., 2011; Löwe et al., 2008). Respondents were asked to rate the presence of symptoms in the past 2 weeks on a 4-point Likert scale ranging from 0 (*not at all*) to 3 (*almost every day*). Higher total scores indicate more severe symptoms of anxiety. The Dutch version of the GAD-7 has been validated before (Donker et al., 2011). Internal consistency in the current study was acceptable (ω = 0.77).

### Data Analyses

A factor analytic approach was adopted to select the items for the SAIL-SF, which is one of the most common and popular item reduction strategies for short-form development (Chiu et al., 2020; Coste et al., 1997; Marsh et al., 2005; Prieto et al., 2003). Factor analyses were performed with Mplus 8.3 (Muthén & Muthén, 2017), all other analyses with IBM SPSS Statistics 28.0.

As the first step in the development of the SAIL-SF, the original factor model of the SAIL was fitted to the data from sample 1 (nurses) and sample 2 (cancer patients) using confirmatory factor analysis (CFA). A correlated seven-factor model was fitted separately in both samples, in which each item was only allowed to load on its own factor and item errors were not allowed to correlate. Robust weighted least square mean and variance (WLSMV) adjusted estimators were adopted to account for the ordinal nature of Likert-type item responses (Brown, 2006; Flora & Curran, 2004; Moshagen & Musch, 2014). Because the chi-square (χ^2^) statistic is overly sensitive to detect misfit in larger samples (Brown, 2006), multiple indices were used to examine model fit, including the Tucker–Lewis Index (TLI), the comparative fit index (CFI), the standardized root mean square residual (SRMR) and the root mean square error of approximation (RMSEA) (Hu & Bentler, 1998). TLI and CFI values ≥ 0.90 and 0.95 were considered indicative of acceptable and good model fit, respectively. For the SRMR and RMSEA, values ≤ 0.10 and 0.08 and ≤ 0.08 and 0.06, respectively, were considered to reflect acceptable and good fit (Browne & Cudeck, 1993; Hu & Bentler, 1999). After fitting the models, the item that best represented each factor in each sample was identified based on its standardized factor loadings in the CFA model (Marsh et al., 2005). Standardized factor loadings were interpreted as adequate when > 0.40 (Costello & Osborne, 2009; Ford et al., 1986).

In the second step, the results from CFAs were shared within an expert team meeting (EB, LF, AV, PtK) to discuss the final item selection based on the previously introduced criteria aiming at a final seven-item scale. The content of the items with the highest factor loading in each sample was evaluated and discussed to avoid sacrificing content coverage by relying exclusively on statistical item characteristics (Coste et al., 1997; Smith et al., 2000). Discussions about factors with different items loading the highest in each sample and their conceptual representativeness for the respective aspect of spiritual well-being were resolved by consensus.

In the third step, exploratory factor analysis (EFA) with varimax rotation was performed on the items selected in step 2 in the first and second sample to evaluate the dimensionality and factor loadings of the 7 items. The number of empirical dimensions underlying the seven items was determined using parallel analysis (PA) (Horn, 1965). Because PA does not work well with categorical data and Mplus does not allow for parallel analyses with WLSMV, maximum likelihood estimation was used which assumes continuous variables. A component was considered relevant if its observed eigenvalue in the actual data exceeded the 95^th^ percentile of its respective eigenvalue generated from 10,000 randomly generated datasets with the same number of items and sample size (Glorfeld, 2016; Schmitt, 2011). As with the CFAs, factor loadings of the EFA model as determined by PA were interpreted as adequate when > 0.40. The internal consistency of the final SAIL-SF items was examined using McDonald’s omega (ω) with 10,000 bootstrapped 95% confidence intervals using the HA method (Hancock & An, 2020) in the SPSS OMEGA macro (Hayes & Coutts, 2020). Coefficients below 0.70 were considered unacceptable, coefficients between 0.70 and 0.79 acceptable, between 0.80 and 0.89 good and greater than 0.90 excellent (Cicchetti, 1994). To examine the equivalence of the SAIL-SF with the original SAIL, Pearson correlations were computed between mean total SAIL-SF and SAIL scores. Because correlations between total SAIL and total SAIL-SF scores based on a single administration of the same instrument will be spuriously inflated, additional corrected correlation coefficients (*r*_c_) were computed that adjust for the shared measurement error due to the same items being present in both the short form and the full version (Levy, 1967). High correlations (i.e., *r* values ≥ 0.90 and *r*_c_ values ≥ 0.70) were considered indicative of substantial overlap between the constructs measured by the full and short form versions of the SAIL.

The unidimensionality, internal consistency, construct validity and incremental validity of the final SAIL-SF were evaluated in sample 3. To confirm unidimensionality, a strict unidimensional CFA model was fitted on the seven SAIL-SF items. As in the development phase of the SAIL-SF, WLSMV estimation was used and the same criteria were used to examine model fit and measurement quality (factor loadings). Next, internal consistency was tested with McDonald’s ω. For convergent validity of the SAIL-SF, it was hypothesized a priori that spiritual well-being scores would be correlated moderately negatively with anxiety and depression (Braam & Koenig, 2019; Garssen et al., 2021; Weber & Pargament, 2014) and strongly positively with emotional, psychological and social well-being (Garssen et al., 2021; Salsman et al., 2015; Smith et al., 2012; van Dierendonck & Mohan, 2007; Visser et al., 2010); Also, it was expected that spiritual well-being would be moderately to strongly correlated with higher positive reinterpretation and growth coping style and perceived generic ability to adapt scores (Newlin et al., 2011; Ivtzan et al., 2011; Schwalm et al., 2022). As per Cohen’s suggestion, correlations > 0.50 were interpreted as strong, correlations between 0.30 and 0.50 as moderate, and correlations < 0.30 as small (Cohen, 1988). For incremental validity of the SAIL-SF (Haynes & Lench, 2003), it was hypothesized that spiritual well-being scores would explain a significant proportion in peoples’ ability to adapt, above and beyond the variance explained by emotional, psychological and social well-being. To test this assumption, a blockwise hierarchical linear regression was performed, regressing generic sense of the ability to adapt scores on emotional, social and psychological well-being (MHC-SF) scores in block 1 and adding spiritual well-being (SAIL-SF) in block 2.

## Results

### Development of the SAIL-SF

#### Confirmatory Factor Analysis of the SAIL-SF in Sample 1 and 2

The correlated seven-factor CFA model demonstrated acceptable (RMSEA) to good (CFI, TLI and SRMR) fit for the original SAIL in the sample of patients with cancer (Table 1). In the nurses' sample, the CFI and SRMR indices suggested adequate fit, whereas the TLI and RMSEA did not meet the defined cutoffs for acceptable model fit. Nonetheless, in both samples all items loaded sufficiently high on their respective factor, supporting the measurement quality of the SAIL (Table 2). Standardized correlations between the seven latent factors ranged between 0.35 and 0.95 in sample 1 and between 0.24 and 0.80 in sample 2 (all *p*’s < 0.001).

**Table 1:** Fit Indices for the Correlated Seven-Factor CFA Model of the SAIL in Sample 1 (Nurses) and Sample 2 (Cancer Patients)

Fit Index	Sample 1 *n* = 458	Sample 2 *n* = 445
χ^2^ (df)	1328.536 (278)	732.805 (278)
CFI	.904	.961
TLI	.888	.954
RMSEA (90% CI)	.091 (.086 – .096)	.061 (.055 – .066)
SRMR	.061	.048

SAIL, Spiritual Attitude and Involvement List, CFA, Confirmatory Factor Analysis, CFI, Comparative Fit Index, TLI, Tucker-Lewis Index, RMSEA, Root Mean Square Error of Approximation, SRMR, Standardized Root Mean Square Residual

**Table 2:** Standardized Factor Loadings for the Correlated Seven-Factor CFA Model for the SAIL in Sample 1 (Nurses) and Sample 2 (Cancer Patients)

Dimension	Item	Item Content	Sample 1	Sample 2
Meaningfulness	4	I know what my position is in life	.612	.616
	12	I experience the things I do as meaningful	**.727**	.792
	17	My life has meaning and purpose	.719	**.841**
Trust	1	I approach the world with trust	.660	.565
	3	In difficult times I maintain my inner peace	.670	.616
	9	Whatever happens, I am able to cope with life	.587	.685
	13	I try to take life as it comes	**.785**	**.787**
Acceptance	6	I accept that I am not in full control over the course of my life	.661	.770
	8	I accept that I am not able to influence everything	.635	.615
	11	I am aware that each life has its own tragedy	.682	.727
	15	I accept that life will inevitably sometimes bring me pain	**.775**	**.817**
Caring for Others	2	It is important to me that I can do things for others	.763	.812
	7	I am receptive to other people's suffering	.709	.715
	16	I try to make a meaningful contribution to society	**.827**	.814
	18	I want to mean something to others	.814	**.893**
Connectedness with Nature	5	The beauty of nature moves me	.837	**.863**
	14	When I am in nature, I feel a sense of connection	**.914**	.825
Transcendent Experiences	19	I have had experiences during which the nature of reality became apparent to me	.756	.758
	20	I have had experiences in which I seemed to merge with a power or force greater than myself	.879	.886
	21	I have had experiences in which all things seemed to be part of a greater whole	**.896**	**.944**
	23	I have had experiences where everything seemed perfect	.672	.652
	25	I have had experiences where I seemed to rise above myself	.813	.800
Spiritual Activities	10	There is a God or higher power in my life that gives me guidance	.701	.793
	22	I talk about spiritual themes with others (themes such as the meaning of life, death or religion)	.756	.767
	24	I meditate or pray, or take time in other ways to find inner peace	**.891**	**.885**
	26	I attend sessions, workshops etc. that are focused on spirituality or religion	.830	.766

Highest factor loadings in each sample are in boldface

#### Item Selection

For the dimensions of trust (item 13), acceptance (item 15), transcendent experiences (item 21), and spiritual activities (item 24) the same items had the highest factor loading in both samples and were considered suitable and representative for the respective aspect of spiritual well-being by the expert team. For the dimension meaningfulness, item 12, which loaded strongest only in the nurses' sample, was preferred due to possible content overlap of item 17 with an item from the psychological well-being dimension of the MHC-SF. For the caring for others dimension, item 16, which again loaded most strongly in the nurses' sample only, was preferred over item 18 as the latter item did not appear to truly grasp the content of caring for others and instead may point more towards a need for affiliation. Finally, for the connectedness with nature dimension item 14 loaded the highest in sample 1, whereas item 5 loaded the highest in sample 2. As the content of the items suggested no clear reason for choosing one over the other, it was decided to select the item that correlated the strongest with the other six selected items. Because item 14 had the highest corrected item-total correlation with the other six items in both sample 1 (*r* = 0.57 vs. 0.46) and sample 2 (*r* = 0.46 vs. 0.41), item 14 was preferred and selected over item 5.

#### Parallel and Exploratory Factor Analysis

**Fig. 1:**
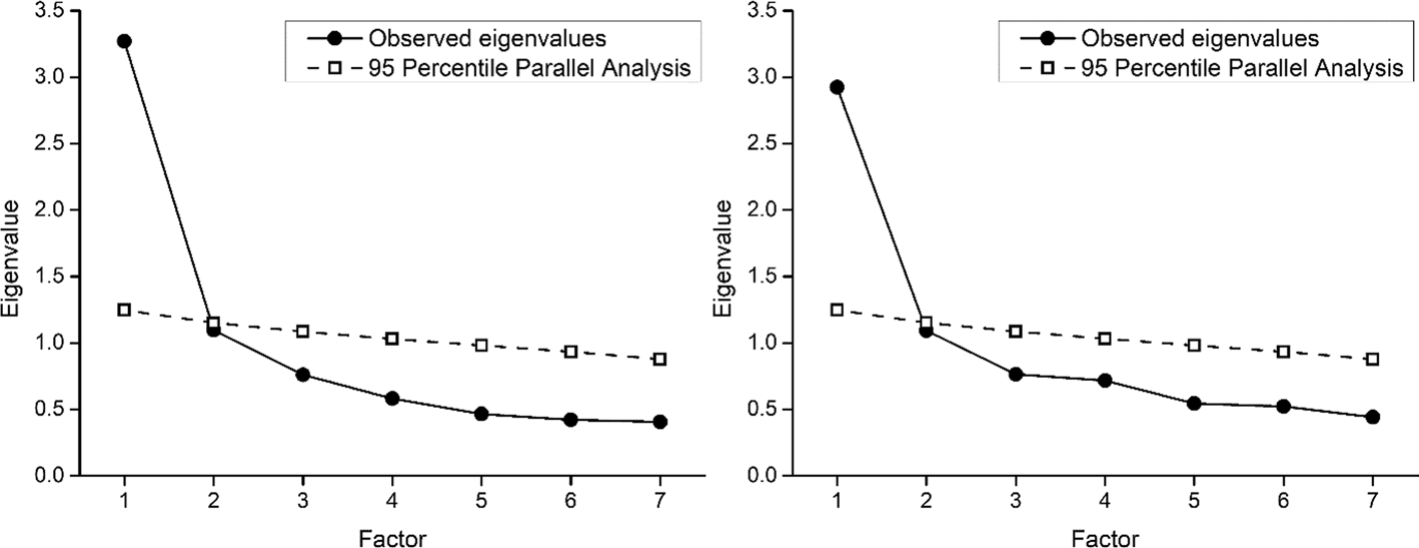
Parallel Analysis of the Final Seven SAIL-SF Items in Sample 1 (Nurses, Left Panel) and Sample 2 (Cancer Patients, Right Panel)

In both samples, parallel analysis showed that only the eigenvalue of the first observed factor was greater than the 95^th^ percentile of the distribution of eigenvalues derived from the random data, suggesting a single meaningul factor underlying the seven items in both samples (Fig. 1). Factor loadings in the single factor solution were adequately high in both sample 1 (ranging from 0.55 for item 7 to 0.73 for item 4) and sample 2 (between 0.47 for item 6 to 0.66 for items 1 and 4).

### Internal Consistency of the SAIL-SF and Equivalence with the SAIL

Internal consistency of the seven selected items for the SAIL-SF was adequate in both sample 1 (ω = 0.79, 95% CI 0.75–0.83) and sample 2 (ω = 0.74, 95% CI 0.69–0.78). Total mean scores of the SAIL-SF were highly correlated with total mean scores of the original version with *r* = 0.93 (*r*_c_ = 0.69) in sample 1 and *r* = 0.91 (*r*_c_ = 0.71) in sample 2. Although the corrected correlation coefficient was slightly below the a-priori defined standard in sample 1, this suggests that both versions measure reasonably similar overall constructs.

### Validation of the SAIL-SF

#### Unidimensionality and Internal Consistency

Assessment of the unidimensionality of the seven-item SAIL-SF in sample 3 showed adequate fit according to the CFI and SRMR, but poor fit according to both the TLI and RMSEA for a strict single-factor CFA model (Table 3). Inspection of the modification indices pointed towards a strong association (modification index = 153) between the error terms of item 1 (I experience the things I do as meaningful) and 4 (I try to make a meaningful contribution to society), likely due to shared error variance caused by the use of the word “meaningful” in both items. Allowing the error terms of this pair of items to correlate (standardized *r* = 0.49, *p* < 0.001) in a subsequent model, resulted in strongly improved and good model fit according to the CFI, TLI and SRMR, although the RMSEA value was still slightly above the cutoff for adequate fit. Standardized factor loadings in the strict unidimensional model were all sufficiently high, ranging from 0.49 for item 7 to 0.89 for item 1 (Table 4), suggesting good measurement quality. The internal consistency of the seven items was good (ω = 0.81, 95% CI 0.76–0.84).

**Table 3:** Fit Indices for the Unidimensional CFA Model of the SAIL-SF

Model	χ^2^ (df)	CFI	TLI	RMSEA (90% CI)	SRMR
Strict unidimensional model (no error correlations)	193.550 (14)	.919	.878	.239 (.210 – .269)	.073
Unidimensional model with error correlation between items 1 and 4	46.805 (13)	.985	.975	.108 (.075 – .0142)	.033

SAIL-SF, Spiritual Attitude and Involvement List-Short Form; CFA, Confirmatory Factor Analysis; CFI, Comparative Fit Index; TLI, Tucker-Lewis Index; RMSEA, Root Mean Square Error of Approximation; SRMS, Standardized Root Mean Square Residual

**Table 4:** Standardized Factor Loadings for the Strict Unidimensional CFA Model for the SAIL-SF

Item	Loading
1. I experience the things I do as meaningful	.882
2. I try to take life as it comes	.688
3. I accept that life will inevitably sometimes bring me pain	.614
4. I try to make a meaningful contribution to society	.871
5. When I am in nature, I feel a sense of connection	.570
6. I have had experiences in which all things seemed to be part of a greater whole	.682
7. I meditate or pray, or take time in other ways to find inner peace	.489

SAIL-SF, Spiritual Attitude and Involvement List-Short Form

#### Convergent Validity

As hypothesized, total SAIL-SF scores were strongly positively associated with all three dimensions of mental well-being as measured with the MHC-SF and moderately negative with PHQ-9 depression scores and GAD-7 anxiety scores (Table 5). Total SAIL-SF scores were also positively correlated with higher COPE positive reinterpretation and growth coping style scores and perceived generic ability to adapt scores, although these correlations were even somewhat stronger than expected.

**Table 5:** Internal consistencies and correlations between the different measures

		Pearson’s *r*							
Variable	ω	1	2	3	4	5	6	7	8
1. SAIL-SF	.81								
2. MHC-SF total	.92	.62							
3. MHC-SF EWB	.85	.55	.86						
4. MHC-SF SWB	.76	.53	.91	.66					
5. MHC-SF PWB	.86	.63	.95	.78	.78				
6. GSAAS	.92	.66	.68	.62	.56	.68			
7. COPE^a^	.87	.66	.56	.48	.49	.57	.63		
8. GAD-7	.81	− .32	− .41	− .41	− .33	− .41	− .41	− .31	
9. PHQ-9	.77	− .43	− .54	− .54	− .44	− .51	− .53	− .29	.61

All correlations significant at *p* < .001

SAIL-SF, Spiritual Attitude and Involvement List-Short Form; MHC-SF, Mental Health Continuum – Short Form; EWB, emotional well-being; SWB, social well-being; PWB, psychological well-being; GSAAS, Generic Sense of Ability to Adapt Scale; COPE, Coping Orientation to Problems Experienced inventory; GAD-7, Generalized Anxiety Disorder scale,. PHQ-9, Patient Health Questionnaire

^a^Subscale positive reinterpretation and growth

#### Incremental Validity

Results of the regression analysis showed that spiritual well-being explained a significant proportion of variance in ability to adapt scores (Δ*R*^2^ = 0.07, *p* < 0.001) above and beyond the variance explained by emotional, psychological and social well-being, supporting the incremental validity of this additional aspect of well-being (Table 6).

**Table 6:** Summary of Blockwise Multiple Hierarchical Regression of Generic Sense of the Ability to Adapt (GSAAS) on Emotional, Social and Psychological Well-being (MHC-SF) and Spiritual Well-being (SAIL-SF)

Variable	*B* (*SE*)	Β	*t*	Δ*R*^2^	Δ*F* (*df*)
Block 1				.52	78.77 (3, 218)***
MHC-SF EWB	1.59 (0.56)	.21	2.85**		
MHC-SF SWB	0.29 (0.58)	.04	0.49		
MHC-SF PWB	4.07 (0.71)	.51	5.74***		
Block 2				.07	37.91 (1, 217)***
MHC-SF EWB	1.19 (0.52)	.16	2.29*		
MHC-SF SWB	0.10 (0.54)	.01	0.18		
MHC-SF PWB	2.67 (0.69)	.34	3.85***		
SAIL-SF	3.29 (0.53)	.36	6.16***		

MHC-SF, Mental Health Continuum – Short Form; EWB, emotional well-being; SWB, social well-being; PWB, psychological well-being

^*^p < .05, **p < .01,***p < .001

## Discussion

The current study aimed to develop and validate a short form of the Spiritual Attitude and Involvement List (SAIL). First, using data of earlier research in large samples of nurses and cancer patients (Van Leeuwen et al., 2015; Visser et al., 2017), a thorough factor-analytic and expert approach was used to select seven items, each representing one of the seven original subscales of the SAIL. It was found that the seven items represented a single meaningful factor in both samples and that the factor loadings of the items were adequately high in both samples. The internal consistency of the seven items was found to be adequate in both samples. Also, the total mean scores of the short and the long form of the SAIL were strongly correlated suggesting that both versions measured similar constructs. Secondly, the newly developed SAIL-SF was validated in a sample of adults participating in a trial assessing the impact of a positive psychology app on mental well-being, in the context of coping with the consequences of the COVID-19 pandemic. When the error terms of two items were allowed to correlate, a good model fit across the various model indexes was found and all items had adequately high factor loadings in a strict unidimensional model and demonstrated good internal consistency. The pattern of moderate and strong correlations of the SAIL-SF with measures of well-being, depression, anxiety, positive reinterpretation, and ability to adapt was generally in line with our expectations, demonstrating convergent validity. Finally, it was found that the SAIL-SF explained an additional 7% of variance in ability to adapt above and beyond the MHC-SF, measuring emotional, psychological, and social well-being, underscoring the incremental validity of spiritual well-being to these other aspects of well-being.

These findings show that the SAIL-SF is a psychometrically adequate brief instrument to assess spiritual well-being in line with the broad perspective of the original SAIL. Based on a nontheistic approach, the SAIL was developed to measure spiritual functioning of people with both a religious and secular background (de Jager Meezenbroek et al., 2012a). The SAIL aims to measure spirituality as the experience of connectedness with oneself, the environment and, the transcendent. The results suggest that the seven items of the SAIL-SF, representing the seven original subscales of the SAIL, meaningfulness, trust, and acceptance (oneself), caring for others and connected with nature (environment) and, spiritual activities and transcendent experiences (transcendent) form a unidimensional and reliable instrument. We found an error-correlation between two items (‘I experience things I do as meaningful' and 'I try to make a meaningful contribution to society’), possibly reflecting an artifact as a result of the use of the word meaningful in both items. A good overall model fit was found, after allowing the error terms of this pair of items to correlate. As the standardized factor loadings of the two items were sufficiently high in the strict unidimensional model and, combined with the good reliability of the SAIL across the three samples, this suggests good overall measurement quality of the SAIL-SF, despite the observed error-correlation.

The finding that spiritual well-being explained additional variance in ability to adapt above and beyond emotional, psychological, and social well-being is relevant. To our best knowledge this is one of the first studies directly comparing spiritual well-being and the other aspects of well-being in relation to adaptation. The ability to adapt can be seen as a vital determinant of health (e.g., Bohlmeijer & Westerhof, 2021; Kashdan & Rottenberg, 2010) and it is important that researchers study the processes underlying adaptation (Diener et al., 1999; Park, 2012). The adaptive value of emotional and psychological well-being has been well documented. Many studies have shown that emotional well-being contributes to health and longevity (Chida & Steptoe, 2008; Diener & Chan, 2011; Lamers et al., 2012). Experimental studies have demonstrated that positive emotions broaden our attention and our behaviour repertoire promoting long-term resources for successful adaptive coping with difficult life events (Fredrickson, 1998, 2001). In a similar vein, psychological well-being is associated with adaptive neurochemical changes and good physical health and reduced mental health issues and resources for adaptive coping such as flexible and creative thinking and pro-social behaviour (Huppert, 2009; Ryff, 2014). Our study shows that spiritual well-being is significantly associated with a sense of the ability to adapt beyond emotional, psychological, and social well-being. This finding is in line with earlier studies showing that spirituality is an essential component of the good life. For example, van Dierendonck (2012) found that in a scenario study that spirituality positively contributed to a well-lived life in terms of desirability and moral goodness. Ivtzan et al. (2013) found a positive association between spirituality and self-actualization, meaning in life and personal growth as key indicators of psychological well-being (Ryff, 1989). The significant correlation between psychological and spiritual well-being we found in our study corroborates the linkage between the two aspects of well-being. However, the moderately high association between the two, in addition to the incremental value of spiritual well-being, suggests that psychological and spiritual well-being are distinct aspects of well-being. Our findings are also in line with a growing body of research demonstrating a relationship between spirituality and adaptive coping. For example, in a recent review, Schwalm et al. (2022) found a moderate association between spirituality and adaptive coping across 34 studies. Recently, Rogers et al. (2022) found a significant positive relationship between spirituality and resilience in a sample of over 700 advanced clinical practitioners coping with the impact of COVID-19 on their professional lives. However, it must be noted that most of these studies are cross-sectional, limiting the possibility to make causal inferences.

We argue that the availability of the SAIL-SF will facilitate further research on spiritual well-being as a universal experience. It has been discussed that the adaptive consequences of spiritual well-being are somewhat understudied in comparison to especially emotional and psychological well-being (Swinton, 2001; van Dierendonck, 2012). Though there is growing evidence for a positive relationship between spiritual well-being and adaptive coping and health, many studies have some important limitations such as selective and smaller samples and being descriptive (Schwalm et al., 2022; Unterrainer et al., 2014). In order to study spiritual well-being as a vital resource for resilience and health and to allow causal inferences, longitudinal studies are warranted, preferably in large, representative samples. In comparison, there is a large number of studies examining the long-term impact of emotional and psychological well-being (e.g., Chida & Steptoe, 2008; Ryff et al., 2015; Keyes et al., 2010; Schotanus et al., 2017). In a similar vein, further research is needed to examine the mechanisms through which spiritual well-being has an impact on health-related conditions (Smith et al., 2012; Unterrainer et al., 2014). Experience sampling is an emerging research design to study within-person processes in the context of daily life and real-world dynamics (Hofmann et al., 2020; Myin-Germeys et al., 2018). It typically requires participants to fill out questions several times a day over a number of days. Therefore, ESM-studies are dependent on the use of a limited number of items. The SAIL-SF may be useful to study spiritual attitudes and interests in daily life and its role in coping with daily stressors. Another interesting line of research is the assessment of spiritual well-being as an outcome of interventions. If future research corroborates the predictive value of spiritual well-being for adaptation, resilience and health, it will be of interest to assess to what extent spiritual well-being can be enhanced in various settings. The impact of interventions on spiritual well-being has been extensively studied in cancer survivors. A recent meta-analysis of 41 trials found small to medium effects of psychosocial interventions on spiritual well-being across studies (McLouth et al., 2020). There is also initial evidence that mindfulness-based programs may promote spiritual well-being (e.g., Carmody et al., 2008; Greeson et al., 2011). However, more research is needed to study the impact of existing and innovative interventions on spiritual well-being. A short form of the SAIL promotes its use in longitudinal panel and experimental studies where there is often strong competition with other instruments and where the total number of items have to be limited to prevent response effects and drop-out due to lack of motivation (Bradburn et al., 2004; Herzog & Bachman, 1981). The SAIL-SF may be particularly relevant when there is a primary interest in spiritual attitudes related to sense of connection with self, environment and transcendent that make sense to religious and non-religious individuals with occasional spiritual experiences.

### Strengths and Limitations

A strength of the current study is that a thorough procedure was followed for both item selection and psychometric evaluation of the SAIL-SF. The development of the SAIL-SF was based on different samples. Also, the evaluation of its factor-structure, reliability and validity was conducted in a different sample to prevent overestimation of its psychometric properties. The inclusion of the MHC-SF, a frequently used similar measure for emotional, psychological, and social well-being facilitated the assessment of the effects of spiritual well-being beyond and over these aspects of well-being with well established relationships with adaptive coping and health. The current study also has limitations. First, the samples of healthcare workers (sample 1, N = 458) and adults with cancer (sample 2, N = 445) that were used to select the most representative items from the original SAIL were rather small for a correlated seven-factor CFA model with 26 items and did not meet the minimum recommended ratio of five observations per freely estimated parameter (sample 1, k = 176; sample 2, k = 165) in either sample (Bentler & Chou, 1987). Therefore, these factor solutions and the exact parameter estimates for the original SAIL should be interpreted with caution. However, the main objective of these CFA models was to identify the items with the highest loading for each factor, and not so much a robust validation of the factor structure of the original SAIL. The final sample used to test the unidimensional CFA model for the seven-item SAIL-SF did exceed the 5:1 ratio of sample size (N = 225) to number of free parameters (k = 42). Second, males and lower educated people were underrepresented in the various samples. This diminishes the generalizability of the findings. Third, we were unable to test several important additional psychometric properties of the SAIL-SF in this study, including its temporal stability (test–retest reliability), sensitivity to change, known-groups validity and concurrent validity against other, already established, spiritual well-being instruments. Additional studies using larger and more diverse samples are needed to collect more evidence of the psychometric soundness of the SAIL-SF, especially regarding its dimensionality. It is also important to note that in the short form each dimension of spiritual interest and attitude is measured with only one item. Researchers or practitioners with an interest in the specific dimensions of the SAIL are recommended to use the full form.

## Conclusion

The current study shows that the SAIL-SF has good psychometric properties, and that spiritual well-being is significantly associated with the ability to adapt over and beyond emotional, psychological, and social well-being. The brevity of the SAIL-SF facilitates the study of spiritual well-being as a universal experience of connectedness to self, environment and transcendent in longitudinal panel, experience-sampling and experimental studies. Replication of the findings in other samples using different constructs for concurrent and convergent validity and study of its sensitivity to change and test–retest reliability are warranted.
